# Global seroepidemiology of pertussis: a systematic review and meta-analysis

**DOI:** 10.3389/fpubh.2026.1807398

**Published:** 2026-06-03

**Authors:** Lulu Meng, Yue Yu, FeiYang Lu, Shijie Gao, Jiang Wu, Zhujiazi Zhang

**Affiliations:** 1 Beijing Key Laboratory of Surveillance, Early Warning and Pathogen Research on Emerging Infectious Diseases, Beijing Center for Disease Prevention and Control, Beijing, China; 2 Beijing Research Center for Respiratory Infectious Diseases, Beijing, China; 3 School of Public Health, Capital Medical University, Beijing, China; 4 School of Public Health, Peking University Health Science Center, Beijing, China

**Keywords:** infection, meta-analysis, pertussis, random-effects models, seroepidemiology, systematic review

## Abstract

**Background:**

Pertussis remains a global health concern despite widespread vaccination. Clinical reporting underestimates true disease burden due to underdiagnosis and asymptomatic cases. Consequently, serological surveillance is essential for accurately assessing infection rates and population immunity.

**Methods:**

We conducted a systematic review and meta-analysis of studies published between January 2014 and October 2024 in PubMed, Web of Science, EMBASE, and the Cochrane Library. Observational studies reporting the seropositivity rate of PT-IgG or recent pertussis infection rate in general populations were included. Pooled estimates were calculated using random-effects models, with subgroup analyses by region, sex, age, and investigation year.

**Results:**

Analyzing 64 studies involving approximately 87,000 participants from 37 countries, the global pooled seropositivity rate was 23.3% (95% CI: 17.2–30.1%), and the recent infection rate was 3.6% (95% CI: 2.4–5.0%). Seropositivity exhibited a trimodal distribution: the first peak in infants aged 1–2 (25.8%), the highest peak in adolescents aged 10–19 (26.2%), a nadir in adults aged 20–39 (17.1%), followed by a tertiary rise in individuals ≥40 years (20.5%). Recent infection rates showed a similar pattern. Regional disparities were significant, with the Americas showing the highest seropositivity (51.2%), while the Eastern Mediterranean Region reported the highest recent infection rate (10.3%). Temporally, seropositivity increased from 18.0% (2011–2015) to 25.9% (2016–2022), whereas recent infection rates declined from 6.4 to 2.7%.

**Conclusion:**

This study confirms the ongoing global circulation of *Bordetella pertussis* and insufficient population immunity. The revealed age- and regional disparities underscore the need to advance pertussis control from childhood immunization toward a precision public health model informed by seroepidemiological data.

## 1 Introduction

Pertussis, a highly contagious acute respiratory disease caused by *Bordetella pertussis*, is one of the world’s major vaccine-preventable infectious diseases (1). According to WHO data, in 2024, 89% of infants worldwide (approximately 115 million) received at least one dose of the combined vaccine for diphtheria, tetanus, and pertussis, and 85% (about 109 million) completed the full three-dose vaccination schedule (2). Although global vaccination efforts have significantly reduced infant mortality, pertussis remains a persistent public health challenge (3). Over the past two decades, countries with high vaccination coverage, including the United States, Australia, and the United Kingdom, have reported a notable resurgence in pertussis cases, giving rise to the phenomenon known as pertussis resurgence (4). Preliminary data from the U.S. Centers for Disease Control and Prevention (CDC) indicate that the number of reported cases in 2024 was more than six times higher than that in 2023 (5).

The resurgence of pertussis is driven by complex factors, including the spread of non-vaccine-type ptxP3 strains represented by multi-drug resistant MT28 clones (6), waning vaccine-induced immunity (7), variations in vaccination strategies, and a transformation in transmission patterns, characterized by adolescent- and adult-to infant transmission during the period of large-scale vaccination (8). Consequently, the true incidence of pertussis is far higher than reported.81% of adult cases only presented with non-febrile paroxysmal cough, with up to 95% of infections undiagnosed and unreported (9, 10).

Therefore, relying solely on case reports makes it difficult to accurately capture the true epidemic intensity and transmission dynamics. Integrating standardized serological surveillance into routine epidemiological practice can effectively identify infections that go undetected clinically (11)—particularly past infections and mild cases—thereby enabling a more comprehensive assessment of disease burden, evaluation of population immunity, monitoring of the durability of vaccine protection, and providing critical scientific evidence for adjusting immunization strategies (12). The WHO suggests incorporating serological studies into the immunization program evaluation system and using the detection of PT-IgG levels to determine pertussis recent infection (3).

Although previous studies have evaluated aspects of pertussis epidemiology or seroprevalence within specific regions or age groups (13–15), a comprehensive global meta-analysis of PT-IgG seroprevalence integrating both seropositivity and recent infection rates across all ages and WHO regions is currently lacking. To address this gap, this study synthesizes the latest global serological evidence through a systematic review and meta-analysis. Specifically, we aimed to estimate pooled seropositivity and recent infection rates, evaluate demographic and temporal trends across age groups and regions, and ultimately inform evidence-based recommendations for optimizing pertussis prevention and control strategies worldwide.

## 2 Methods

### 2.1 Registration

The study protocol was developed based on PRISMA guidelines (16) and is registered on PROSPERO, CRD42024626623. No changes have been made from the protocol in the conduct of this systematic review.

### 2.2 Search strategy

We searched for articles published between January 1, 2014, and October 1, 2024 in the following databases: PubMed, Web of Science, EMBASE, and the Cochrane Library. Search strings were constructed in collaboration with a medical librarian, used the search terms ‘Pertussis’, ‘Whooping Cough’, ‘Antibodies’, ‘Pertussis Toxin’, ‘PT-IgG’, ‘FHA-IgG’, ‘Seroepidemiology’, ‘Seroprevalence’, ‘Seropositivity rate’, and ‘Recent infection rate’ and written in English (Supplementary Table S1).

### 2.3 Study selection

The inclusion criteria were as follows: (1) observational studies, such as cross-sectional studies, epidemiological surveys, and surveillance studies; (2) described the general population; and (3) the outcome included the seroprevalence of PT-IgG. Studies that met the following criteria were excluded: (1) studies on clinical characteristics, treatments, molecular biology, risk factors, popular science lectures, newspaper articles, and literature reviews; (2) duplicate studies or studies with overlapping participants; (3) those with insufficient data to calculate the outcomes in terms of seroprevalence; and (4) those in which the study population comprised confirmed or clinically suspected cases of pertussis.

NoteExpress (3.2.0.7629) was used to eliminate duplicates. Two reviewers (SJG, FYL) independently screened the studies based on the inclusion criteria described above. Disagreements were resolved by consensus or consultation with a third reviewer (LLM).

### 2.4 Data extraction

We developed Excel templates for data extraction, performing the methodological quality assessment, subgroup analysis. Review team members (SJG, FYL and LLM) extracted the data and assessed risk of bias of included studies. In the event of missing or inaccurate data, the authors of the original articles were contacted.

For each eligible study, we extracted information on: (1) study characteristics, including study design, study subjects, sample design, sampling frame and response situation, (2) laboratory testing details, such as reagents, antibody detection assays and results definition, (3) antibody outcomes, specifically total antibody levels and interval distributions. Value was calculated by the reviewers when it was not explicitly reported but could be estimated from the available data. Authors were contacted if additional study information was required.

Data on country-specific pertussis vaccination schedules were obtained from the WHO website (17). Thresholds for defining PT-IgG seropositivity and recent infection were extracted from each included study, as these criteria vary according to the commercial ELISA kits used or the in-house assay protocols (18).

### 2.5 Methodological quality assessment

A quality assessment was performed for included studies based on the criteria developed from the guidelines for the evaluation of incidence studies (19, 20). Studies were given a score of 0 to 8 based on the degree to which they fulfilled eight criteria related to the assessment, the quality of the statistical analysis, and the extent to which the sample population represented the population at large.

### 2.6 Statistical analysis

Stata (version 16.0) was used to perform all of the statistical calculations in this meta-analysis. Using the Freeman-Turkey Double Arcsine Transformation (FTT), data were combined and estimated for the pooled seroprevalence of PT-IgG and the recent infection rate of pertussis using the metaprop command (21). The seropositivity rate and 95% confidence intervals (CIs) of the subgroups were analyzed by region, sex, age, and investigation year. The *I*^2^ value was used to assess the heterogeneity of the included studies (22). Pooled estimates were calculated using random-effects models. Publication bias was assessed using Egger’s regression test, with *p* < 0.05 used to indicate evidence of publication bias (23). Leave-one-out sensitivity analysis was performed to assess the stability of the pooled seropositive rate of PT-IgG and the recent infection rate of pertussis.

The generation of global maps were performed using R software (version 4.2.1). Country-level geographic boundaries were obtained from the rworldmap and rworldxtra packages. The sf package was used for spatial data processing. Data management and analysis were conducted with the dplyr package. Figures were created using the ggplot2 package.

## 3 Results

### 3.1 Search results

**Figure 1:**
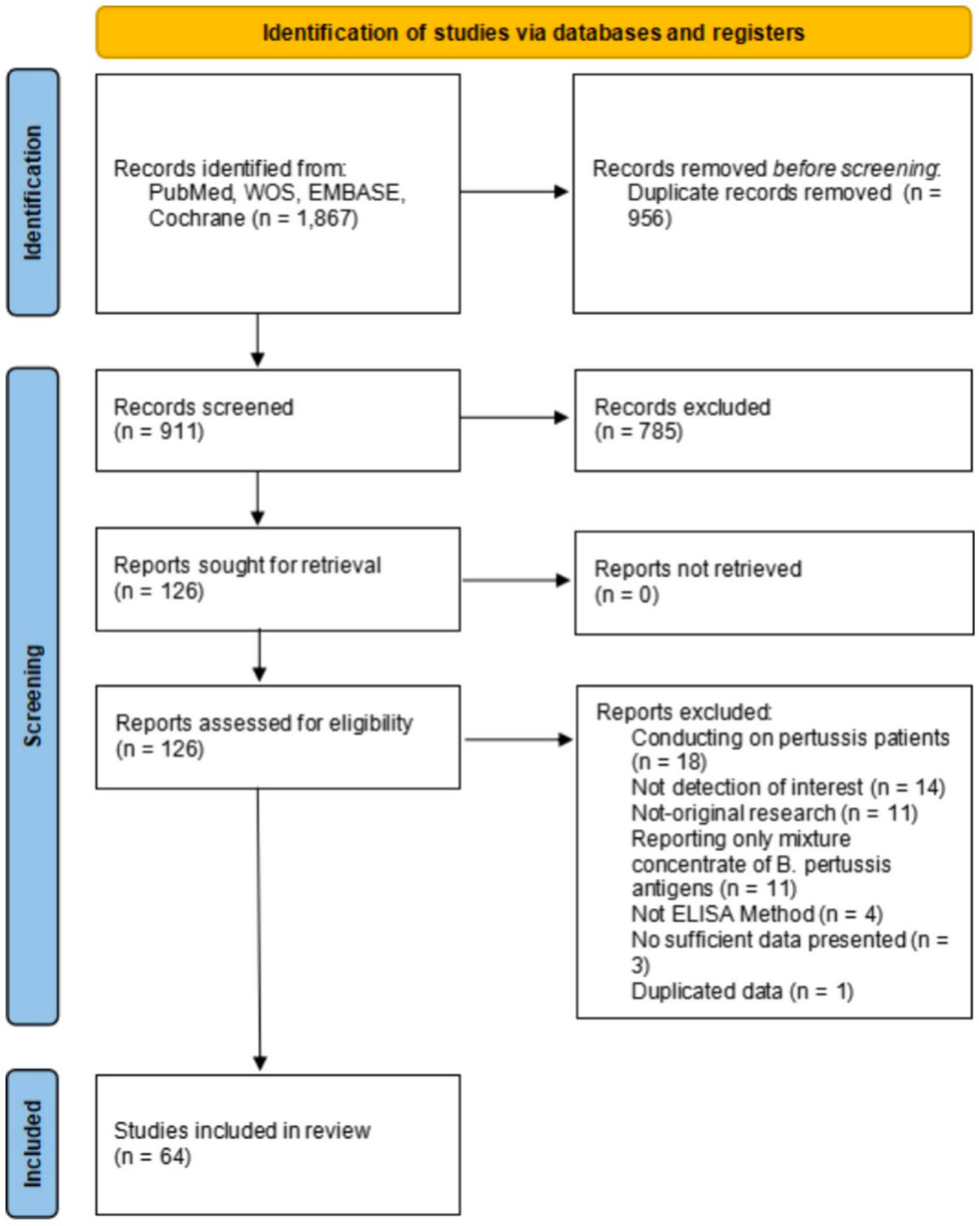
Study search and inclusion. The process of literature screening, inclusion, and exclusion followed the PRISMA guidelines.

A total of 1,867 articles were initially identified from the four databases, 126 full-text articles were selected for further evaluation. After excluding 62 studies, 64 original studies met our inclusion criteria for meta-analysis (Figure 1, Supplementary Table S2).

### 3.2 Study characteristics

All study data were collected between 2005 and 2022, originated from 37 countries across six regions. The regional distribution of studies was as follows: Western Pacific (*n* = 25), European (*n* = 18), Eastern Mediterranean Region (*n* = 8), South-East Asian (*n* = 7), African (*n* = 2), and the Americas (*n* = 4). The target populations were diverse, including general population (*n* = 16), children or adolescents (*n* = 17), adults (*n* = 15), pregnant women (*n* = 9) and healthcare workers (*n* = 7). Methodologically, 57 studies used commercial ELISA kits with positive and recent infection cut-off values defined by the kit instructions. For these commercial kits, the positive cut-off value ranged from 5 IU/mL to 125 IU/mL (with equivalent thresholds expressed in EU/mL, FDA-U/mL, U/mL, NTU, or ESEN units/mL), and the recent infection cut-off value ranged from 40 IU/mL to 125 IU/mL (or the corresponding values in other units). Seven studies employed in-house developed methods (Supplementary Table S3), the positive cut-off was 75 IU/mL and the recent infection cut-off ranged from 30 IU/mL to 125 IU/mL. The quality scores of the included studies ranged from 4 to 8, with a median score of 6 (Supplementary Table S4). Among the 37 countries included in the analysis, 26 utilized aP in their childhood routine immunization programs, while 11 used wP. Beyond routine childhood immunization. Additionally, 18 countries implemented programs for other specific groups, including pregnant women (12 countries) and adults (9 countries), with 4 countries recommending a booster dose every decade (Supplementary Table S5).

### 3.3 Seropositivity and recent infection rates of PT-IgG

**Figure 2:**
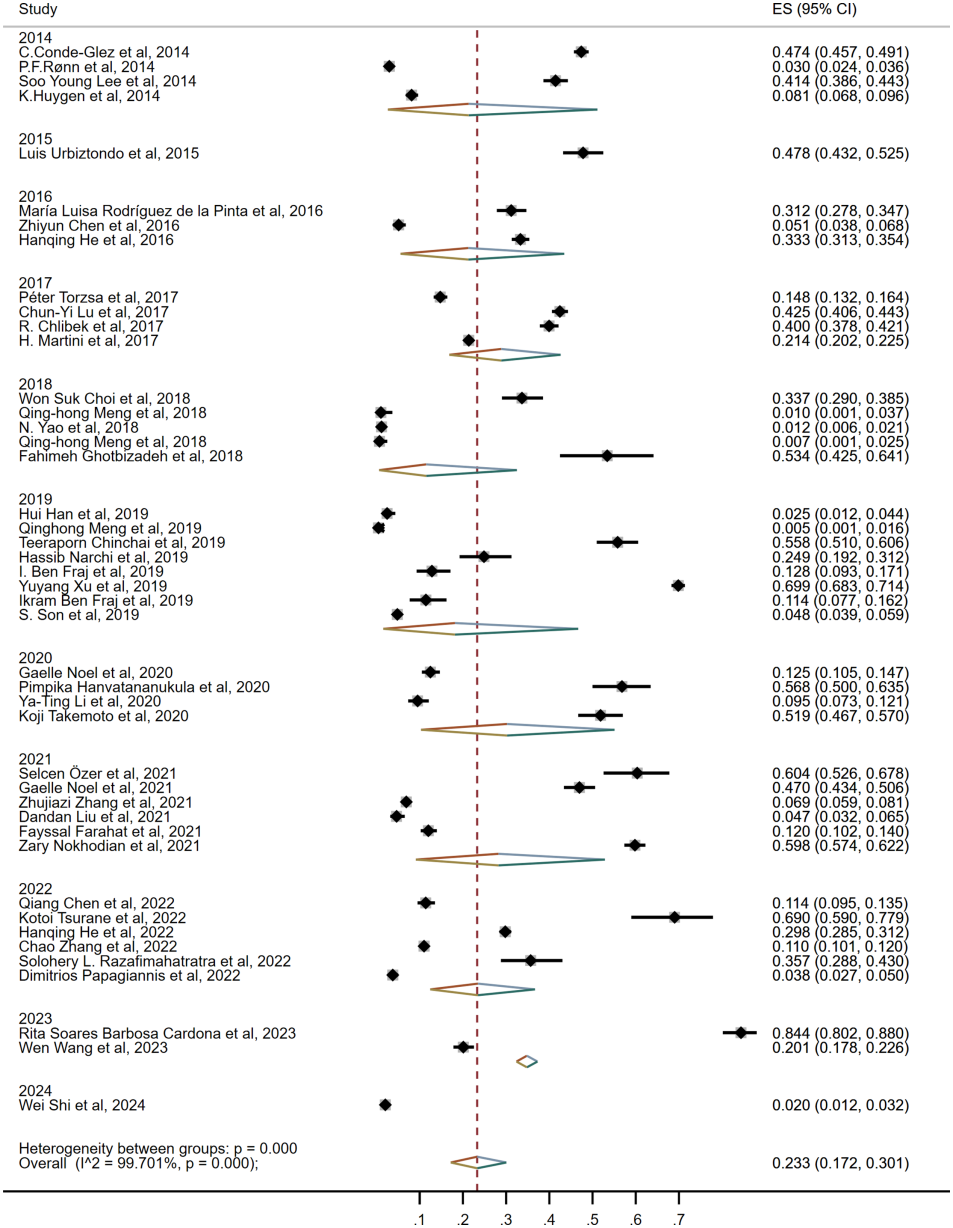
Forest plot of overall estimates of seropositivity rate of PT-IgG.

**Figure 3:**
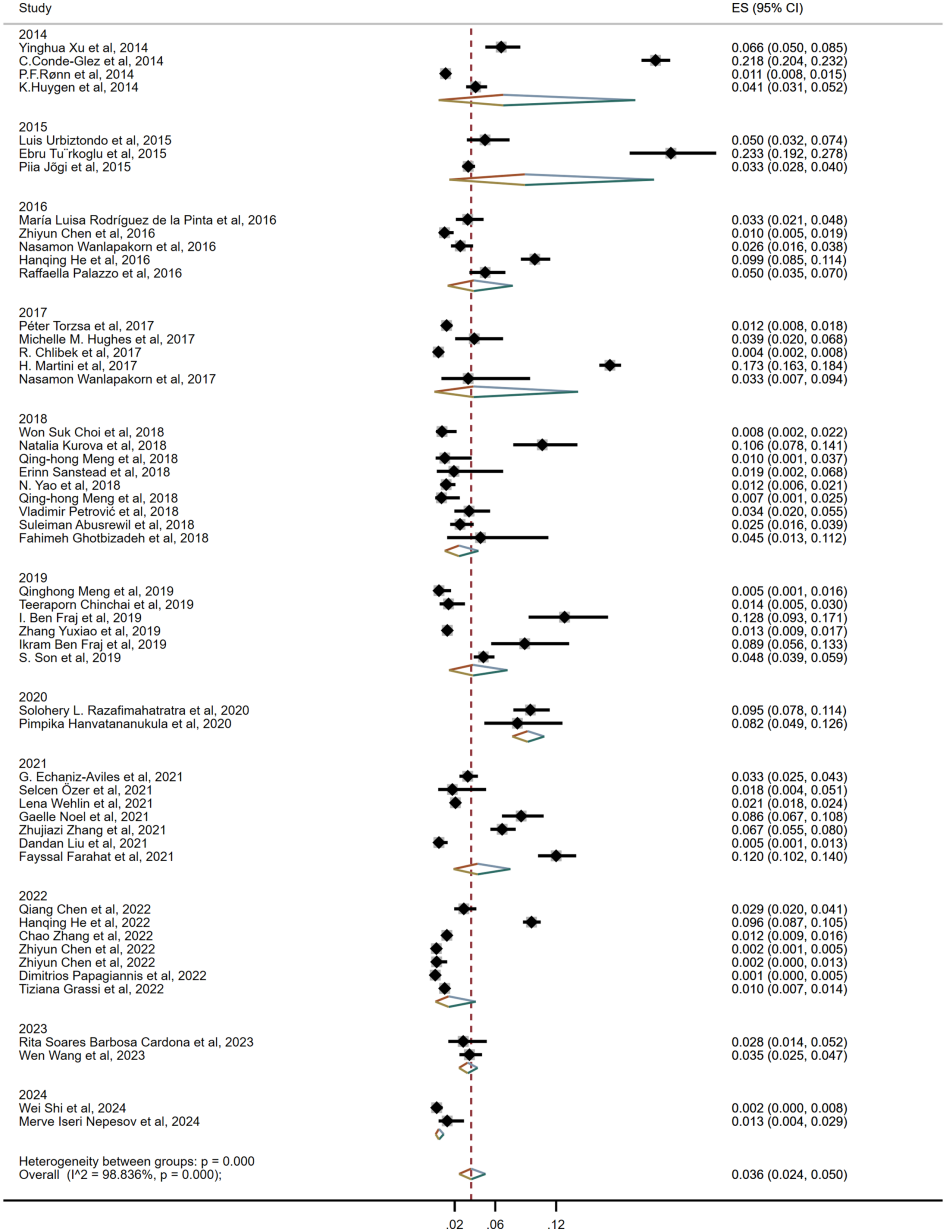
Forest plot of overall estimates of recent infection rate of pertussis.

The meta-analysis indicated that the studies had statistically significant heterogeneity (*I*^2^ > 90%), thus the pooled estimate of the seropositivity rate and the recent infection rate were calculated using random effects models. The pooled seropositivity rate estimate was 23.3% (95% CI: 17.2–30.1%) (Figure 2) and the pooled estimate of the recent infection rate was 3.6% (95% CI: 2.4–5.0%) (Figure 3).

No significant differences in seropositivity or recent infection rates were observed between males and females (*p* = 0.869 and *p* = 0.788, respectively) (Table 1).

**Table 1:** Subgroup analysis for seropositivity rate of PT-IgG and recent infection rate of pertussis.

Variables	Seropositivity rate					Recent infection rate				
	No. of data points	Sample size	Rate (95% CI), %	*I*^2^, %	*p*-value	No. of data points	Sample size	Rate (95% CI), %	*I*^2^, %	*p*-value
Overall	44	56,810	23.3 (17.2, 30.1)	99.7		52	72,903	3.6 (2.4, 5.0)	98.8	
Region*										
WPR	28	30,474	15.5 (8.9, 23.6)	99.7	<0.001	25	27,038	2.2 (1.1, 3.6)	97.7	<0.001
EUR	9	16,345	22.5 (12.4, 34.6)	99.6		17	34,026	3.7 (1.7, 6.3)	99.2	
EMR	7	4,644	24.7 (9.2, 44.8)	99.5		4	1828	10.3 (7.7, 13.2)	59.9	
SEAR	5	1,466	21.5 (3.1, 50.0)	99.3		8	2,761	4.1 (2.6, 5.9)	74.7	
AMR	2	3,881	51.2 (49.6, 52.9)	—		4	5,381	6.0 (0.1, 19.1)	99.4	
AFR	1	185	35.7 (28.8, 43.0)	—		2	1869	6.0 (5.0, 7.2)	—	
Sex										
Male	26	18,860	21.4 (13.1, 31.0)	99.6	0.869	15	12,468	2.2 (0.7, 4.6)	97.8	0.788
Female	30	20,987	22.4 (14.5, 31.5)	99.5		12	11,380	1.9 (0.8, 3.4)	96.1	
Age group										
<1y	14	1824	24.4 (12.9, 37.8)	96.9	0.869	7	1,235	7.1 (0.0, 23.9)	98.3	0.063
1–2y	7	1,145	25.8 (5.6, 53.5)	98.7		4	799	6.0 (0.0, 25.6)	97.7	
3-4y	7	1,498	22.8 (4.8, 48.6)	98.9		5	1,698	5.6 (1.3, 12.3)	95.7	
5–9y	14	2,833	22.2 (8.7, 39.5)	98.9		10	2,258	6.9 (2.3, 13.5)	96.0	
10-19y	27	6,516	26.2 (14.9, 39.3)	99.2		16	4,474	6.7 (3.2, 11.2)	96.2	
20-39y	29	7,220	17.1 (10.7, 24.7)	98.3		31	17,862	2.3 (1.2, 3.6)	95.0	
≥40y	43	11,219	20.5 (15.0, 26.6)	98.0		28	9,627	5.3 (3.1, 8.1)	96.5	
Investigation year										
2005–2010	4	8,141	21.4 (1.6, 54.9)	99.9	0.465	6	11,598	6.4 (0.8, 16.5)	99.7	0.416
2011–2015	13	20,779	18.0 (10.5, 27.0)	99.6		19	32,739	3.9 (2.1, 6.1)	98.8	
2016–2022	29	27,890	25.9 (17.1, 35.8)	99.7		29	28, 566	2.7 (1.5, 4.2)	97.8	

—, No data; CI, confidence interval.

*Western Pacific Region (WPR), European Region (EUR), Eastern Mediterranean Region (EMR), South-East Asian Region (SEAR), African Region (AFR), Region of the Americas (AMR).

Seropositivity rates exhibited a trimodal distribution, with the first peak in the 1–2y age group (25.8, 95% CI: 5.6, 53.5) and the highest peak in the 10–19y group (26.2, 95% CI: 14.9–39.3), declining to the lowest point in the 20–39y group (17.1, 95% CI: 10.7–24.7), before rising again in those aged ≥40y (20.5, 95% CI: 15.0–26.6). A similar fluctuating pattern was observed for recent infection rates, with the highest rate in the <1y group (7.1, 95% CI: 0.0–23.9), followed by subsequent peaks in the 5–9y (6.9, 95% CI: 2.3–13.5) and 10–19y (6.7, 95% CI: 3.2–11.2) groups, and the lowest rate in the 20–39y group (2.3, 95% CI: 1.2–3.6) (Table 1).

### 3.4 Seropositivity and recent infection rates by region

**Figure 4:**
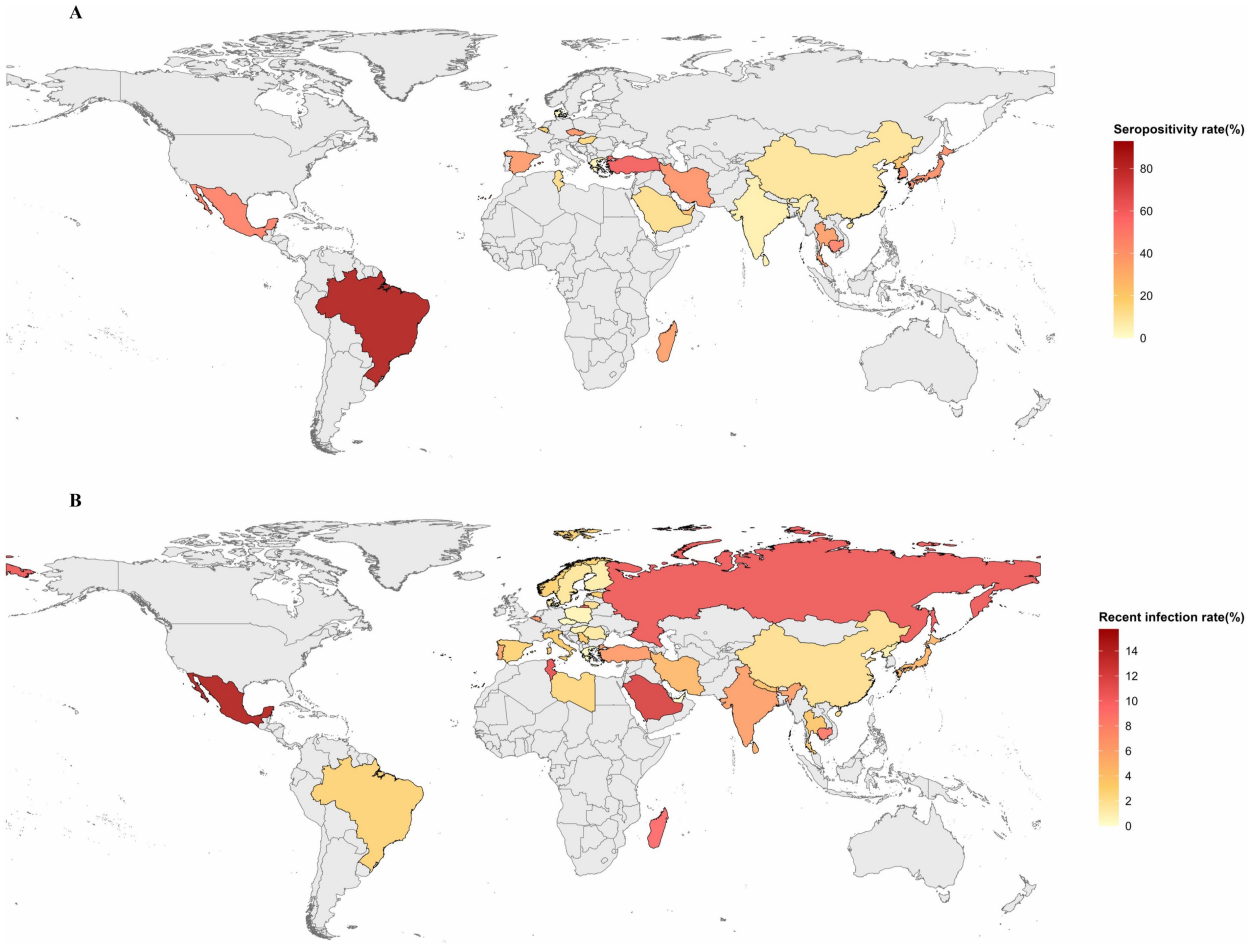
Spatial distribution of **(A)** PT-IgG seropositivity and **(B)** recent pertussis infection rates across 37 countries. The intensity of the color corresponds to the magnitude of the rate, with darker shades indicating higher values. Due to limitations in data access, the gray areas represent regions with no available data.

Seropositivity and recent infection rates varied significantly across regions (*p* < 0.001) (Table 1). Figure 4 and Supplementary Table S6 exhibited the spatial distribution of seropositivity and recent infection rates, covering a total of 37 countries.

**Figure 5:**
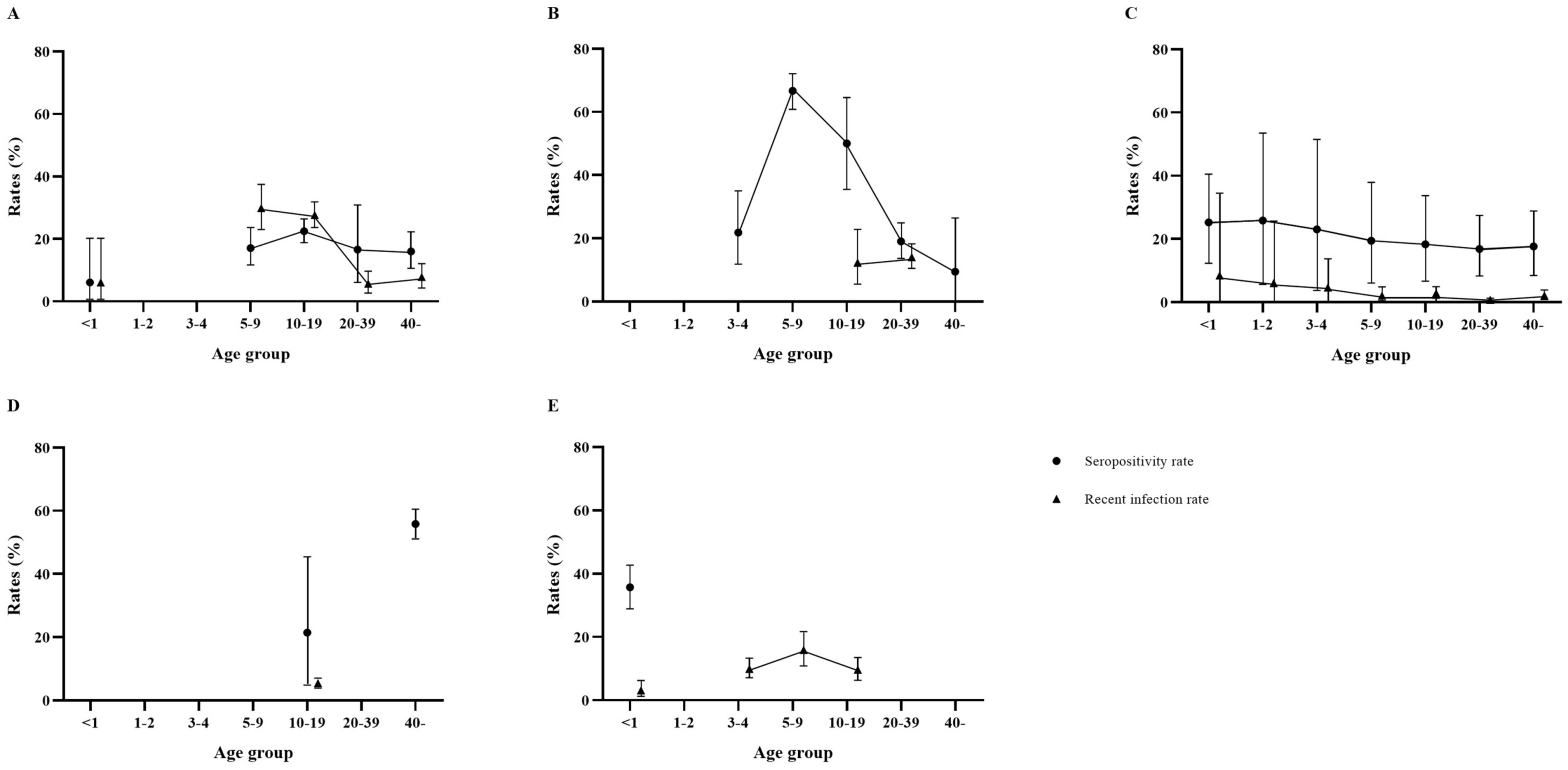
The seropositivity rate of PT-IgG and recent pertussis infection rates by region: **(A)** Europe; **(B)** Eastern Mediterranean; **(C)** Western Pacific; **(D)** South-East Asia; **(E)** Africa.

Seropositivity rates were high in the South East Asia Region among older adults (≥40 years: 55.8%), while the Eastern Mediterranean Region showed pronounced peaks among school aged children (5–9 years: 66.7%) and adolescents (10–19 years: 50.0%). In contrast, Europe and the Western Pacific Region exhibited more balanced age distributions, with maximum seropositivity rates of 22.5 and 25.8%, respectively (Figure 5).

Recent infection rates were persistently elevated among adolescents and adults in the Eastern Mediterranean (10–39 years: >12%), whereas Europe displayed a distinct peak in school aged children (5–9 years: 29.9%). The African Region showed a concentration in the pediatric population (5–9 years: 15.9%), and the Western Pacific Region maintained low recent infection rates across all age groups (≤8.4%) (Figure 5).

### 3.5 Seropositivity and recent infection rates by year

Seropositivity rates were the lowest in 2011–2015 (18.0, 95% CI: 10.5–27.0) and the highest in 2016–2022 (25.9, 95% CI: 17.1–35.8). Recent infection rates declined over time, from 6.4% (95% CI: 0.8–16.5) in 2005–2010 to 2.7% (95% CI: 1.5–4.2) in 2016–2022 (Table 1).

**Figure 6:**
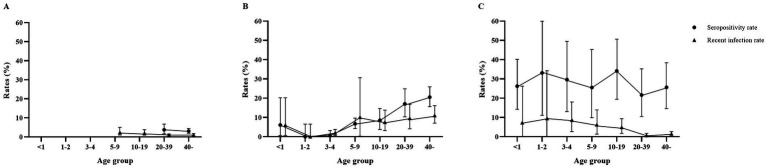
The seropositivity rate of PT-IgG and recent pertussis infection rates by year: **(A)** 2005–2010; **(B)** 2011–2015; **(C)** 2016–2022.

During 2005–2010, seropositivity remained low (3.0–3.7%) among adults aged 20 years and above. The 2011–2015 period witnessed an increase in seropositivity across all ages, particularly among adolescents and adults (8.5–20.5%). Notably, the data from 2016 to 2022 exhibited an elevated rates in young children, reaching 26.2% in infants under 1-year-old and 33.1% in 1-2-year-old, while recent infection rates declined in older adolescents and adults (Figure 6).

### 3.6 Publication bias and sensitivity analysis

Although the Egger’s regression test suggested potential publication bias (*p* < 0.05), the sensitivity analyses were performed by removing individual studies one at a time and recalculating the pooled statistics. The overall findings showed no significant changes in direction, indicating the stability of the results (Supplementary Tables S7, S8).

## 4 Discussion

This systematic review and meta-analysis synthesized serological evidence from nearly 87,000 participants across 37 countries, providing a comprehensive, systematic depiction of the global pertussis seroepidemiological landscape from 2005 to 2022, offering insights into the current risk profiles and epidemiological patterns worldwide.

The pooled seropositivity rate was 23.3% and the recent infection rate was 3.6%, collectively indicating a composite immunity profile shaped by both vaccination and natural infection, and confirming the ongoing circulation of *Bordetella pertussis* within the population. However, the overall immunity level remains far below the theoretical herd immunity threshold of approximately 80% required to interrupt transmission (R0 = 15–17), suggesting a persistently high epidemic risk and ongoing active transmission globally (24, 25).

The observed seropositivity rates exhibited high statistical heterogeneity, with point estimates varying widely across studies. Rather than indicating methodological limitations, this variability likely reflects genuine differences in global exposure histories and immune backgrounds (1). Moreover, inconsistencies in detection thresholds—such as variations in the cut-off values used by different commercial ELISA kits or in-house assays—directly influenced the calculation of seropositivity rates (26). We employed a random-effects model for pooled estimation to account for this inherent diversity. Subsequent subgroup analyses further revealed regional and age-related disparities, confirming the uneven global distribution of pertussis immunity.

Pertussis seroepidemiology exhibited a trimodal age distribution. Seropositivity rates peaked in toddler (25.8%) and adolescence (26.2%), declined to a nadir in young adulthood, and rebounded in individuals aged ≥40 years. Recent infection rates followed a similar fluctuating pattern, with the highest rate observed in the <1-year-old age group (7.1%). These findings align with observations from multiple regions globally, revealed heterogeneity in susceptibility and transmission dynamics across age groups (27–30).

The high seropositivity in infancy is attributable primarily to maternal antibodies, primary vaccine responses, and a high inherent susceptibility to infection at this age (31, 32). The second peak in adolescence strongly suggests significant waning of vaccine-induced immunity over time (33), a vulnerability compounded by the group’s active social interactions, which increase the infection and transmission risk (34). Of particular note, the high rate of infection in school-aged children highlights a critical vulnerability in immunization programs in countries that lack a preschool booster dose following completion of the primary infant series, identifying this period as a key window for booster vaccination (35). The serological pooled seropositivity rate was likely multifactorial, resulting from waning immunity of acellular vaccines (36), lower natural exposure, and the exclusion of adults from routine booster programs in most countries (37). This immunity gap may allow adults to become reservoirs for asymptomatic or paucisymptomatic infection, serving as a significant f transmission threat to vulnerable infants (38). Therefore, building upon the success of primary immunization, a strategic expansion of prevention policy is warranted. Implementing a preschool booster is crucial to mitigate the transmission in early childhood. Furthermore, extending vaccination programs to encompass adolescents and adults is essential to bridge this immunity gap, establishing a life-course immunization barrier that ultimately provide more effective protection for infants (39, 40).

Seroprevalence of pertussis showed substantial regional disparities, resulting from the complex interplay between local immunization strategies and distinct epidemiological contexts. In Europe, characterized by high vaccine coverage and low disease incidence, the elevated antibody levels observed among school-aged children coincided temporally with booster vaccination schedules (27). This pattern serves as evidence of the expected population-level immunological response following vaccination. In regions such as the Eastern Mediterranean, despite the existence of immunization programs, persistently high antibody levels across multiple age groups likely reflect an inability of vaccine-induced protection to fully prevent infection under ongoing community transmission pressure. The serological profile in these areas is consequently a superposition of vaccine-mediated immunity and natural infection. Conversely, the Western Pacific Region exhibited comparatively lower population-wide antibody levels (41). The lack of adolescent and adult booster strategies in this region, unlike in Europe, has created a pronounced immunity gap within older age groups. The serologically revealed divergence in immunological landscapes across regions necessitates consideration for implementing tailored immunization strategies, guided by the integration of serological and conventional epidemiological surveillance (42).

Temporally, seropositivity rates for pertussis have shown an upward trend over the past two decades, particularly in adults. Globally, especially in middle- and high-income countries, pertussis vaccine coverage has been consolidated (43), and the gradual implementation of booster immunization strategies for adolescents, pregnant women, and other specific groups has directly contributed to increased population antibody levels. As a result, the rate of recent infections has shown a corresponding decline, aligning with the global reduction in disease burden (44).

This study is subject to certain limitations. First, despite the application of random-effects model, significant statistical heterogeneity (*I*^2^ > 90%) was observed across most analyses, indicating substantial variation in seroprevalence estimates that may stem from unmeasured study-level factors (45). Second, the serological criteria for defining both seropositivity and recent infection, while based on established cut-off values, vary across commercial kits and in-house assays, potentially affecting the comparability of results between studies (46). Third, our subgroup analyses for certain regions (e.g., SEAR, AFR, AMR) and specific age groups were based on a limited number of data points, which may affect the precision and generalizability of these estimates. Finally, as a serological study, we cannot definitively distinguish between antibody responses induced by natural infection and those from vaccination, particularly in age groups with recent immunization (47).

## 5 Conclusion

This systematic review provides critical serological evidence that augments clinical surveillance, confirming the ongoing global circulation of *B. pertussis* and indicating that population immunity remains insufficient to interrupt transmission, with age-specific and regional disparities shaped by immunization strategies and transmission dynamics. Therefore, the core strategy for pertussis control should evolve from a reliance on childhood primary immunization toward a precision public health model that integrates a life-course vaccination schedule, such as developing recommendations on infant and maternal immunization and adolescent boosters, aligned with the WHO Strategic Advisory Group of Experts on Immunization (SAGE) framework. Additionally, tailoring vaccination schedules to regional seroepidemiological patterns and integrating seroepidemiological data into surveillance systems are critical for guiding targeted vaccination policies and advancing precision public health approaches for pertussis control.

## Data Availability

The original contributions presented in the study are included in the article/Supplementary material, further inquiries can be directed to the corresponding author.
