# Supplementary material for: Global seroepidemiology of pertussis: a systematic review and meta-analysis
Source: Front Public Health. 2026 Jun 3;14:1807398. doi: 10.3389/fpubh.2026.1807398 (PMC13272359; doi:10.3389/fpubh.2026.1807398)
Supplement: Supplementary file 1 [file Supplementary_file_1.docx]

Supplementary Material

**Supplementary Table 1.**

All measles cases that occurred during the outbreak in Mie Prefecture, 2017-18. For the Vaccination History column, a value of 1 means vaccinated once, 2 means vaccinated twice, 0 means unvaccinated, and 10 means status unknown .

| Case  No | Sex | Age | Prefecture of residence | Vaccination history | Onset date | Date reported | Place of  infection | Generation |
| --- | --- | --- | --- | --- | --- | --- | --- | --- |
| 1 | M | 20 | Mie | 0 | January 3, 2019 | January 7, 2019 | camp | 1 |
| 2 | M | 10 | Mie | 0 | January 4, 2019 | January 8, 2019 | camp | 1 |
| 3 | M | 10 | Mie | 0 | January 5, 2019 | January 8, 2019 | camp | 1 |
| 4 | M | 10 | Mie | 0 | January 5, 2019 | January 9, 2019 | camp | 1 |
| 5 | M | 10 | Mie | 0 | January 8, 2019 | January 10, 2019 | camp | 1 |
| 6 | M | 20 | Mie | 0 | January 7, 2019 | January 10, 2019 | camp | 1 |
| 7 | M | 10 | Mie | 0 | January 6, 2019 | January 10, 2019 | camp | 1 |
| 8 | M | 10 | Mie | 0 | January 7, 2019 | January 10, 2019 | camp | 1 |
| 9 | F | 10 | Mie | 0 | January 7, 2019 | January 11, 2019 | camp | 1 |
| 10 | M | 10 | Mie | 1 | January 7, 2019 | January 11, 2019 | camp | 1 |
| 11 | F | 10 | Mie | 0 | January 8, 2019 | January 11, 2019 | camp | 1 |
| 12 | F | 20 | Mie | 0 | January 6, 2019 | January 11, 2019 | camp | 1 |
| 13 | F | 10 | Mie | 1 | January 8, 2019 | January 11, 2019 | camp | 1 |
| 14 | F | 10 | Mie | 1 | January 5, 2019 | January 11, 2019 | camp | 1 |
| 15 | M | 10 | Mie | 0 | January 5, 2019 | January 11, 2019 | camp | 1 |
| 16 | M | 10 | Mie | 0 | January 9, 2019 | January 11, 2019 | camp | 1 |
| 17 | M | 10 | Mie | 0 | January 8, 2019 | January 12, 2019 | camp | 1 |
| 18 | F | 10 | Mie | 0 | January 10, 2019 | January 12, 2019 | camp | 1 |
| 19 | F | 10 | Mie | 0 | January 5, 2019 | January 12, 2019 | camp | 1 |
| 20 | M | 10 | Mie | 1 | January 9, 2019 | January 14, 2019 | camp | 1 |
| 21 | F | 20 | Mie | 0 | January 5, 2019 | January 15, 2019 | camp | 1 |
| 22 | M | 10 | Mie | 0 | January 7, 2019 | January 15, 2019 | camp | 1 |
| 23 | F | 20 | Mie | 0 | January 3, 2019 | January 15, 2019 | camp | 1 |
| 24 | M | 20 | Mie | 0 | January 7, 2019 | January 15, 2019 | camp | 1 |
| 25 | F | 30 | Mie | 1 | January 16, 2019 | January 16, 2019 | family | 2 |
| 26 | F | 30 | Mie | 0 | January 14, 2019 | January 16, 2019 | family | 2 |
| 27 | F | 10 | Mie | 0 | January 16, 2019 | January 18, 2019 | family | 2 |
| 28 | M | 10 | Mie | 0 | January 16, 2019 | January 18, 2019 | family | 2 |
| 29 | F | 20 | Mie | 1 | January 18, 2019 | January 18, 2019 | hospital | 2 |
| 30 | F | 1-4 | Mie | 0 | January 18, 2019 | January 19, 2019 | camp2 | 2 |
| 31 | F | 0 | Mie | 0 | January 15, 2019 | January 21, 2019 | hospital | 2 |
| 32 | M | 10 | Mie | 2 | January 20, 2019 | January 21, 2019 | school | 2 |
| 33 | F | 10 | Mie | 10 | January 20, 2019 | January 21, 2019 | family | 2 |
| 34 | M | 10 | Mie | 10 | January 20, 2019 | January 21, 2019 | family | 2 |
| 35 | M | 40 | Mie | 10 | January 21, 2019 | January 21, 2019 | camp2 | 2 |
| 36 | F | 30 | Mie | 10 | January 19, 2019 | January 22, 2019 | hospital | 2 |
| 37 | F | 20 | Mie | 1 | January 20, 2019 | January 22, 2019 | school | 2 |
| 38 | F | 30 | Mie | 10 | January 22, 2019 | January 23, 2019 | hospital | 2 |
| 39 | M | 10 | Mie | 2 | January 19, 2019 | January 23, 2019 | school | 2 |
| 40 | M | 20 | Mie | 0 | January 18, 2019 | January 24, 2019 | unknown | 2 |
| 41 | F | 10 | Mie | 2 | January 23, 2019 | January 24, 2019 | school | 2 |
| 42 | M | 10 | Mie | 1 | January 21, 2019 | January 25, 2019 | school | 2 |
| 43 | M | 10 | Mie | 2 | January 22, 2019 | January 26, 2019 | school | 2 |
| 44 | M | 40 | Mie | 10 | January 22, 2019 | January 26, 2019 | unknown | 2 |
| 45 | M | 5-9 | Mie | 0 | January 28, 2019 | January 28, 2019 | family | 3 |
| 46 | M | 5-9 | Mie | 0 | January 28, 2019 | January 28, 2019 | family | 3 |
| 47 | M | 20 | Mie | 0 | January 16, 2019 | January 26, 2019 | family | 2 |
| 48 | F | 20 | Mie | 0 | January 28, 2019 | January 29, 2019 | family | 3 |
| 49 | F | 20 | Mie | 2 | January 30, 2019 | February 1, 2019 | hospital | 3 |
| 54 | M | 20 | Shizuoka | 10 | January 19, 2019 | January 20, 2019 | camp2 | 2 |
| 55 | M | 20 | Wakayama | 10 | December 28, 2018 | December 31, 2018 | index | 0 |
| 56 | M | 10 | Wakayama | 10 | January 6, 2019 | January 7, 2019 | family | 1 |
| 57 | F | 20 | Wakayama | 10 | January 9, 2019 | January 10, 2019 | family | 1 |
| 58 | M | 50 | Wakayama | 10 | January 19, 2019 | January 21, 2019 | family | 2 |
| 59 | F | 20 | Gifu | 1 | January 9, 2019 | January 10, 2019 | camp | 1 |
| 60 | M | 20 | Gifu | 0 | January 10, 2019 | January 11, 2019 | camp | 1 |
| 61 | M | 10 | Gifu | 0 | January 10, 2019 | January 11, 2019 | camp | 1 |
| 62 | F | 20 | Gifu | 0 | January 20, 2019 | January 21, 2019 | camp2 | 2 |
| 63 | F | 20 | Gifu | 0 | January 21, 2019 | January 22, 2019 | camp2 | 2 |
| 64 | M | 20 | Aichi | 0 | January 4, 2019 | January 7, 2019 | camp | 1 |
| 65 | M | 20 | Aichi | 0 | January 18, 2019 | January 19, 2019 | family | 2 |
| 66 | M | 20 | Aichi | 2 | January 19, 2019 | January 21, 2019 | unknown | 2 |

# Supplementary Table 2.

Measles cases reported in Osaka prefecture in January 2019.

| No. | Date of Onset | District | Sex | Age Group | Vaccination Status* |
| --- | --- | --- | --- | --- | --- |
| 1 | January 2, 2019 | Osaka City | Female | 40 | 10 |
| 2 | January 3, 2019 | Toyono Town | Male | 40 | 0 |
| 3 | January 4, 2019 | Osaka City | Female | 40 | 0 |
| 4 | January 7, 2019 | Osaka City | Male | 10 | 10 |
| 5 | January 7, 2019 | Osaka City | Male | 20 | 10 |
| 6 | January 9, 2019 | Osaka City | Male | 10 | 1 |
| 7 | January 11, 2019 | Osaka City | Female | 20 | 10 |
| 8 | January 12, 2019 | Toyono Town | Female | 30 | 1 |
| 9 | January 15, 2019 | Toyono Town | Female | 10 | 0 |
| 10 | January 15, 2019 | Toyono Town | Male | 10 | 0 |
| 11 | January 15, 2019 | Osaka City | Male | 10 | 2 |
| 12 | January 17, 2019 | Toyono Town | Female | 10 | 10 |
| 13 | January 17, 2019 | Senshu Town | Female | 20 | 2 |
| 14 | January 18, 2019 | Toyono Town | Male | 10 | 1 |
| 15 | January 18, 2019 | Toyono Town | Female | 30 | 0 |
| 16 | January 18, 2019 | Toyono Town | Female | 30 | 0 |
| 17 | January 19, 2019 | Toyono Town | Female | 10 | 0 |
| 18 | January 20, 2019 | Osaka City | Female | 30 | 10 |
| 19 | January 20, 2019 | Osaka City | Male | 30 | 0 |
| 20 | January 20, 2019 | Osaka City | Female | 60 | 10 |
| 21 | January 20, 2019 | Osaka City | Male | 30 | 10 |
| 22 | January 21, 2019 | Osaka City | Female | 30 | 10 |
| 23 | January 21, 2019 | Mishima Town | Male | 30 | 0 |
| 24 | January 22, 2019 | Kitakawachi Town | Female | 10 | 2 |
| 25 | January 23, 2019 | Toyono Town | Female | 10 | 2 |
| 26 | January 23, 2019 | Osaka City | Male | 30 | 10 |
| 27 | January 23, 2019 | Senshu Town | Male | 10 | 2 |
| 28 | January 24, 2019 | Osaka City | Female | 40 | 2 |
| 29 | January 25, 2019 | Toyono Town | Female | 20 | 2 |
| 30 | January 25, 2019 | Toyono Town | Male | 30 | 10 |
| 31 | January 25, 2019 | Osaka City | Male | 0-9 | 2 |
| 32 | January 27, 2019 | Toyono Town | Female | 0-9 | 2 |
| 33 | January 30, 2019 | Sakai City | Male | 20 | 1 |
| 34 | January 31, 2019 | Toyono Town | Male | 0-9 | 0 |
| 35 | January 31, 2019 | Osaka City | Male | 40 | 0 |
| 36 | January 31, 2019 | Osaka City | Male | 30 | 1 |

*10 for vaccination status represents unknown history of vaccination
